# Musculotendon adaptations and preservation of spinal reflex pathways following agonist‐to‐antagonist tendon transfer

**DOI:** 10.14814/phy2.13201

**Published:** 2017-05-03

**Authors:** Mark A. Lyle, T. Richard Nichols, Elma Kajtaz, Huub Maas

**Affiliations:** ^1^ School of Biological SciencesGeorgia Institute of TechnologyAtlantaGeorgia; ^2^ Department of Human Movement SciencesFaculty of Behavioural and Movement SciencesVrije Universiteit AmsterdamMOVE Research Institute AmsterdamAmsterdamThe Netherlands

**Keywords:** Proprioceptive feedback, regeneration, skeletal muscle, spinal reflex, tendon transfer

## Abstract

Tendon transfer surgeries are performed to restore lost motor function, but outcomes are variable, particularly those involving agonist‐to‐antagonist muscles. Here, we evaluated the possibility that lack of proprioceptive feedback reorganization and musculotendon adaptations could influence outcomes. Plantaris‐to‐tibialis anterior tendon transfer along with resection of the distal third of the tibialis anterior muscle belly was performed in eight cats. Four cats had concurrent transection of the deep peroneal nerve. After 15–20 weeks, intermuscular length and force‐dependent sensory feedback were examined between hindlimb muscles, and the integrity of the tendon‐to‐tendon connection and musculotendon adaptations were evaluated. Three of the transferred tendons tore. A common finding was the formation of new tendinous connections, which often inserted near the original location of insertion on the skeleton (e.g., connections from plantaris toward calcaneus and from tibialis anterior toward first metatarsal). The newly formed tissue connections are expected to compromise the mechanical action of the transferred muscle. We found no evidence of changes in intermuscular reflexes between transferred plantaris muscle and synergists/antagonists whether the tendon‐to‐tendon connection remained intact or tore, indicating no spinal reflex reorganization. We propose the lack of spinal reflex reorganization could contribute the transferred muscle not adopting the activation patterns of the host muscle. Taken together, these findings suggest that musculotendon plasticity and lack of spinal reflex circuitry reorganization could limit functional outcomes after tendon transfer surgery. Surgical planning and outcomes assessments after tendon transfer surgery should consider potential consequences of the transferred muscle's intermuscular spinal circuit actions.

## Introduction

Tendon Transfer surgery is commonly performed to improve or restore lost motor function due to conditions such as cerebral palsy or traumatic injuries to the spinal cord, peripheral nerves and muscles. The biomechanical principle of tendon transfer is straightforward. A donor muscle‐tendon insertion is changed to a new location so as to mimic the mechanical function of a host muscle or to address spastic joint positional deformities (Brand 1993; Friden and Lieber 2002). For example, the rectus femoris tendon is transferred to act as a knee flexor in patients with cerebral palsy to reduce stiff knee gait (Asakawa et al. 2004) and the flexor carpi ulnaris tendon is transferred to the extensor carpi radialis brevis tendon to address wrist flexion deformity (Smeulders and Kreulen 2006). Despite the clear mechanical goals of tendon transfer surgeries, the functional outcomes are variable and unpredictable (Smeulders and Kreulen 2007; van Alphen et al. 2013).

There are a number of possible reasons for the variability in functional outcomes following tendon transfer surgery. Aside from surgical technique and challenges achieving fixation at the most adequate fiber lengths (Friden et al. 2000), there is some evidence that the intended mechanical action of tendon transfers can be compromised either through existing myofascial connections or due to scar tissue formation after surgery (Smeulders and Kreulen 2007). For example, functional benefits observed after transfer of the rectus femoris to act as a knee flexor were assumed to result from its intended knee flexor action. However, it has been shown that transferred rectus femoris muscles retain a knee extensor action and, thus, the functional benefit appears more likely due to reduced knee extension capacity rather than enhanced knee flexion (Riewald and Delp 1997; Asakawa et al. 2004). This outcome was best explained by connective tissue linkages between the rectus femoris and the other knee extensors. A similar outcome was also found in a recent study following agonist‐antagonist tendon transfer in the rat (Maas and Huijing 2012b). These examples support the need for further examination of the potential force shielding consequences of postoperative musculotendon adaptations in response to tendon transfer surgery.

In addition to the biomechanical goal of tendon transfers, appropriate task‐dependent temporal activation of transferred muscles is requisite for successful outcomes. Several reports indicate transferred muscles fail to adopt the activation patterns of the host muscle during motor actions, particularly when the transferred muscle is a direct antagonist (Sperry 1945; Forssberg and Svartengren 1983; Illert et al. 1986; Slawinska and Kasicki 2002). Retraining appears most effective in humans and primates, and even then muscular activity can sometimes revert to the patterns exhibited before the transfer (Sperry 1945, 1947), suggesting that new patterns of activity are not readily established through plasticity at spinal or brainstem levels of control but instead by conscious effort. In the case of locomotion, extensive reorganization of pattern generator and proprioceptive feedback circuitry would appear necessary for correct rhythmic patterning. It is important to emphasize that muscles are linked by proprioceptive feedback, and differences exist in the distribution and strength of intermuscular length and force feedback within muscle groups (e.g., extensors) and more clear differences exist between extensor and flexor muscles (Eccles et al. 1957a, 1957b; Harrison et al. 1983; Bonasera and Nichols 1994; Wilmink and Nichols 2003). While it has been speculated that sensory feedback from muscle spindles and Golgi tendon organs may be capable of reorganizing spinal networks after tendon transfer in an immature nervous system (Slawinska and Kasicki 2002), there has been no direct investigation of whether spinal reflex circuitry is modified after tendon transfer surgery. The failure of a transferred muscle to adopt the host muscle activation patterns (see above described studies) could persist despite changes in spinal reflex circuitry, or could be explained in part by the absence of such changes.

The overall purpose of this study was to evaluate the potential influence of two factors that could limit functional outcomes after tendon transfer (i.e., musculotendon adaptations and lack of sensory feedback reorganization). The primary aim was to examine whether proprioceptive feedback is reorganized to mimic that of the host muscle after agonist‐antagonist tendon transfer surgery in the hindlimb of the cat. We hypothesized that a possible mismatch between usual temporal activation of the transferred muscle and its new mechanical activation of sensory feedback could lead to reorganization of spinal reflex pathways. Because the host muscle (ankle flexor) and transferred muscle (ankle extensor) used in this study are known to have different intermuscular projections mediated by muscle spindles and Golgi tendon organs, signs of reflex reorganization would be indicated by the transferred muscle exhibiting intermuscular actions characteristic of the host muscle. In addition, we evaluated for the presence of musculotendon adaptations after tendon transfer that could prevent and/or limit the transferred muscle from acting in its new role due to mechanical shielding.

We encountered several surprising findings, and challenges, that should be noted at the outset. Intermuscular spinal reflex circuitry and musculotendon adaptations were examined in four cats 15–20 weeks after undergoing plantaris (PLANT) to tibialis anterior (TA) tendon transfer with partial resection of the TA muscle. Interestingly, the TA tendon regrew to its original insertion in two of four cats despite partial muscle resection. Because regrowth of a functioning TA muscle could reduce the need for the transferred PLANT spinal circuitry to reorganize, we completed four additional PLANT‐to‐TA transfer surgeries along with surgical transection of the deep peroneal nerve. An additional challenge we encountered was complete tearing of the PLANT‐to‐TA tendon connection in three cats and thin connections in two cats. The tendon‐to‐tendon connection failures, that is, effectively a tenotomy, provided the opportunity to evaluate the effects of tenotomy on intermuscular spinal reflex circuitry. In light of the challenges noted, we found a strong tendency for the transferred PLANT muscle to establish connections to adjacent tissue and regrowth toward its original calcaneal insertion. Moreover, we found no evidence of spinal reflex reorganization, whether the tendon‐to‐tendon connection remained intact or tore. These findings suggest that musculotendon plasticity and lack of spinal reflex circuitry to reorganize could limit functional outcomes after tendon transfer surgery.

## Methods

All procedures were completed in accordance with guidelines from the National Institutes of Health and approved by the Georgia Institute of Technology Institutional Care and Use Committee. The experiments were performed on eight purpose‐bred female cats ranging from 2.7 to 5 kg (Table 1). The hypotheses tested in this study are based on data collected during a terminal experiment completed between 15 and 20 weeks after tendon transfer surgery (Table 1).

**Table 1:** Subject characteristics and description of musculotendon adaptations

	TT1	TT2	TT3	TT4	TT‐N1	TT‐N2	TT‐N3	TT‐N4
Mass (kg)	3.8	2.7	4.8	3.6	3.2	3.6	3.45	3.3
Nerve cut					DP	DP	DP	DP
Weeks post surgery	15.6	16	19	19	17	18	20	20
Ankle fixation angle	110	110	110	110	110	110	110	90
PLANT‐to‐TA Connection	Intact	Intact	Intact‐slack	Intact‐thin band	Torn	Torn	Intact‐thin band	Torn
Regrowth	a	b	c	a, b	a	b	a, b	a, b

TT, tendon transfer with partial TA muscle resection; TT‐N, tendon transfer with partial TA muscle resection and deep peroneal nerve transection DP, deep peroneal nerve; a, PLANT‐to‐calcaneus; b, TA‐to‐TA insertion; c, PLANT extensive connections toward calcaneus and adjacent tissues.

### Surgical Procedures for tendon transfer

**Figure 1:**
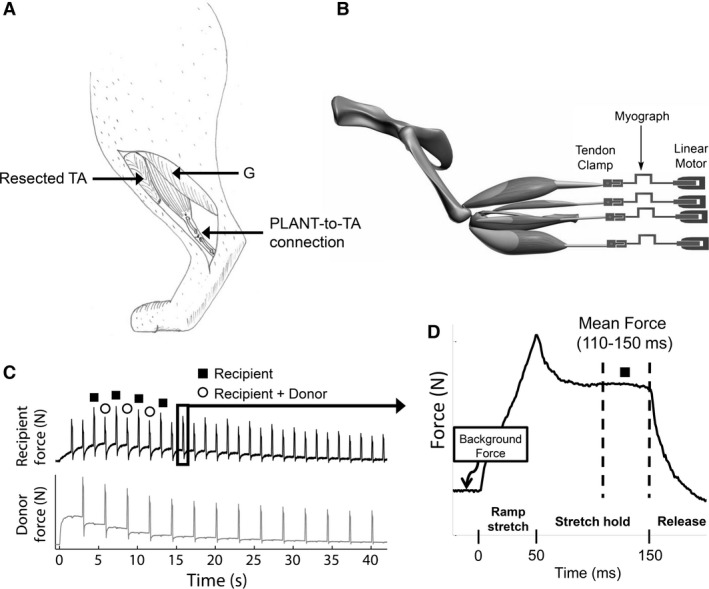
(A) Cartoon of completed PLANT‐to‐TA tendon transfer (medial view) and (B) schematic of experimental set‐up depicting hindlimb muscles detached from their distal insertions and attached in series with myographs and linear motors. During the experiment, two muscles from each limb were connected to separate linear motors and the bone segments were rigidly fixed. (C) Force profiles in response to the ramp stretch‐hold‐release during a crossed extension reflex. In this example, the recipient force is lower when the donor muscle is stretched at the same time consistent with intermuscular force feedback. (D) Example of a single force profile illustrating the mean force time point used for all analyses. TA, tibialis anterior; G, gastrocnemius; PLANT, plantaris

All tendon transfer surgeries were performed on the right hindlimb with cats under general anesthesia using aseptic conditions in a surgical suite. A schematic of the PLANT‐to‐TA tendon transfer is shown in Figure 1A. A longitudinal incision was made from the inferior margin of the popliteal fossa to the calcaneus. The crural fascia was also cut longitudinally to the calcaneus but the medial and lateral fascial bands were kept intact. The longitudinal cut of the crural fascia was repaired after the tendon transfer procedure was finished since the crural fascia is known to influence hindlimb kinematics in the cat (Stahl and Nichols 2014). The PLANT tendon was separated from the Achilles tendon and cut at the level of the calcaneus. The PLANT muscle belly was separated from the lateral gastrocnemius approximately 2/3 of the way along its proximal course with blunt dissection. The PLANT tendon was then pulled under the medial crural fascia band toward the TA insertion. A second incision was made extending from the TA insertion onto the medial aspect of the first metatarsal to its muscle belly. Blunt dissection was used to isolate the tendon and muscle belly from adjacent structures. The TA muscle was transected with electrocautery proximal to the proximal end of the distal aponeurosis, resecting approximately the distal third of its muscle belly. The TA tendon was pulled underneath the transverse crural and cruciate ligaments, then mobilized toward the PLANT tendon and surgically connected using the Pulvertaft method (Pulvertaft 1956). This was accomplished by weaving the TA tendon through the PLANT tendon three to four times with each tendon intersection secured with a mattress suture using 4‐0 silk. The TA tendon was pulled taut to remove slack prior to suture fixation with the ankle joint at 110 degrees in seven cats and 90 degrees in one cat (Table 1). The fixation angle was chosen to correspond with the ankle joint angle achieved during midstance during slope walking (Gregor et al. 2006; Maas et al. 2007) in an effort to mitigate overstretching the transferred muscle (Friden and Lieber 2002). Any free tendon flaps were either trimmed and/or extra sutures were added in an effort to achieve a tendon repair with smooth edges. Fascial layers and the skin were closed with 5‐0 Vicryl suture (Ethicon, Somerville, NJ) and Tissuemend II (VPL, Phoenix, AZ) as needed. No postoperative bracing or casting was used. In an effort to reduce loading of the tendon‐to‐tendon connections, activity was restricted in each cat for 6–8 weeks by keeping them in a large gang cage (59 × 29 × 54 inches) (*n *=* *6) or on the floor of a 4 × 7 foot room (*n *=* *2, TT‐N3, TT‐N4). The cats were then returned to the cat colony with unrestricted activity.

In addition to the tendon transfer, four of eight cats (TT‐N1, 2, 3, and 4) underwent denervation of the ankle and toe extensors (e.g., tibialis anterior and extensor digitorum longus muscles). This was done to deter regrowth of the tibialis anterior tendon (see results). The denervation was completed by making an incision approximately 7–8 cm starting 2 cm distal to the fibular head oriented in line with the biceps femoris muscle fibers. The common peroneal nerve was identified using blunt dissection and a small cut was made through the crural fascia. The peroneal nerve was followed distally so as to identify its branching into deep and superficial peroneal nerves, respectively. The deep peroneal nerve was cut with sharp scissors. A notable dorsiflexion response upon transection was noted in all cats. A small portion of the nerve was resected so as to deter reinnervation. Fascia and skin were closed as described above. Upon awaking from anesthesia, each of the cats exhibited a notable foot drop.

### Surgical procedures for terminal reflex experiment:

The procedures used for evaluating intermuscular reflex circuitry have been reported previously in detail (Ross and Nichols 2009). After a surgical plane of isoflurane gas anesthesia was achieved, tracheal intubation was performed and 1–3% isoflurane used thereafter to maintain deep anesthesia. A cannula was inserted into an external jugular vein to administer saline during the experiment. Adequate anesthesia was confirmed during the experiment by absence of withdrawal reflexes. Heart rate, respiratory rate, oxygen saturation, expired carbon dioxide, and core body temperature were monitored during all procedures. A heating pad was used to maintain core body temperature at 37°C.

The head was fixed in a stereotaxic frame, the abdomen was supported by a sling, and the hindlimbs were rigidly fixed. Hindlimb fixation was achieved using threaded rods in the proximal (just distal to the greater trochanter) and distal femur (femoral condyle) connected by a bar fixed rigidly to the support frame. A separate threaded rod stabilized the proximal tibia, and the distal tibia was stabilized with an ankle clamp.

The anatomy of the distal hindlimb was examined to identify structural adaptations in response to the tendon transfer. After skin was reflected and gross muscle and tendon appearances noted, the PLANT, TA, gastrocnemius (G), soleus (SOL), flexor hallucis longus (FHL), and extensor digitorum longus (EDL) muscles were dissected in both the right and left hindlimbs. Muscles were carefully separated free from adjacent tissues so as to minimize mechanical (myofascial) coupling (Maas and Sandercock 2010) while being careful to preserve their nerve and vascular supplies. On both limbs, the FHL and EDL tendons were cut near their distal insertions, and the G and SOL tendons were separated with small bone chips preserved from the calcaneus. On the uninvolved left limb, the PLANT tendon was cut at the level of the calcaneus, and the TA tendon insertion was removed with a small bone chip from the medial aspect of the first metatarsal. On the right experimental limb, the PLANT and TA tendinous tissues were handled on an individual basis depending on the status of the PLANT‐to‐TA tendon‐to‐tendon connection. For cats with an intact repair, the distal insertion of the transferred PLANT (originally that of TA) was removed with a bone chip from the medial cuneiform if possible. In cases of torn tendon‐to‐tendon connections, the remaining PLANT tendinous connective tissue bands were dissected free to the greatest extent possible. Sufficient tissue from the PLANT was available in all cats for spinal reflex testing. TA tendinous tissues regrew toward its insertion in some cats and so this tissue was cut distally when possible (see results). To eliminate mechanical interactions with surrounding structures, any connective tissue adhesions between the PLANT and TA were carefully freed from adjacent tissues. The distal tendons and/or their tendinous connective tissue bands of all muscles were attached to custom made tendon clamps. The tendon clamps were connected to strain gauge myographs in series with linear motors as shown in Figure 1B.

A standard precollicular decerebration was completed in all cats (Silverman et al. 2005). This involved a vertical transection starting at the anterior margin of the superior colliculus. All brain matter rostral to the transection was removed. Gelfoam and cotton were placed in the cranium to control bleeding. After the decerebration, isoflurane anesthesia was titrated down over approximately 5–30 min and withdrawn. At the end of the experiment, the animals were euthanized with either an overdose of concentrated pentobarbital (Euthasol) or potassium chloride solution after reanesthetizing per guidelines from the American Veterinary Medical Association.

### Data acquisition

Intermuscular spinal reflex pathways were examined using a ramp‐hold‐release muscle stretch protocol. The details of the hardware and software used have been described previously (Wilmink and Nichols 2003; Ross and Nichols 2009; Lyle et al. 2016). In brief, the protocol involved 2 mm muscle stretches with a 50 msec ramp, 100 msec hold and 50 msec release. The muscle stretches for a given trial were applied in a 2‐state alternating pattern with a stretch repetition frequency of 0.7 Hz for a duration of 30–40 sec (i.e., ~10–15 repetitions per state). In state 1, a single muscle referred to as the recipient was stretched. In state 2, the recipient muscle was stretched simultaneously with another muscle referred to as the donor muscle. Muscle stretch repetitions were applied with the recipient and donor muscles' background forces set at a constant ~1–3 N, or over a range of background forces by eliciting a crossed extension reflex. The crossed extension reflex was elicited by stimulating the tibial nerve just proximal to the medial malleolus with a nerve cuff or hook electrode (0.1 msec pulses, 40 Hz, 20‐40 sec duration). Typically, forces in the contralateral extensor muscles increase rapidly, plateau and slowly decay toward the original 1–3 N background force over a 20–40 s time period (Fig. 1C).

### Data analysis and statistics

All data were analyzed using custom programs written in Matlab (Mathworks, Natick, MA). To account for varying background forces and identify the relative force change in response to applied stretches, a baseline force vector for each stretch repetition in a trial was subtracted from the raw force profiles prior to analyses. The baseline force vectors used to remove the background force offset were determined using linear interpolation from stretch onset to a point 900 msec after stretch onset (Ross and Nichols 2009; Lyle et al. 2016). Stretch repetitions that had a sudden background force change precluding an accurate baseline force vector determination were eliminated from analysis, as were spontaneous force responses unrelated to muscle stretch or tibial nerve stimulation during a trial.

The dependent variable was the background force subtracted recipient muscle forces in response to the ramp‐hold‐release stretches. The average muscle force for a period 110–150 msec after stretch onset (i.e., last 40 msec of hold phase) was used to test all hypotheses in this study (Fig. 1D). The force values during this period have previously been shown to illustrate well intermuscular sensory feedback effects from a donor muscle onto recipient muscle (Nichols 1999; Lyle et al. 2016).

To examine the influence of tendon transfer on intermuscular reflex pathways attributed to muscle spindle and Golgi tendon organs, the analysis focused on evaluating whether the PLANT and TA acting as a donor influenced force output in a recipient muscle differently after tendon transfer (i.e., did PLANT elicit intermuscular effects expected of TA). We additionally evaluated whether PLANT acting as recipient was influenced by other muscles after the tendon transfer in a manner similar to that expected of TA. This was accomplished by comparing the force profiles from state 1 (recipient muscle stretched only) and state 2 (recipient muscle stretched at same time as donor) in the left intact and the right transferred muscles quantitatively using the late epoch (i.e., 110–150 msec period after stretch onset) force magnitude. For trials with stretch repetitions recorded with stable background forces, Wilcoxon rank‐sum tests were used to determine whether the recipient muscle forces recorded when stretched alone (state 1) were different from that when stretched pairwise with the donor muscle (state 2). The relative percent differences between recipient muscle forces obtained in states 1 and 2 were calculated to allow for across limb and cat comparisons.

For trials with muscle stretch repetitions applied during the crossed extension reflex, a population of force responses for state 1 and state 2 were recorded over a range of background forces. The populations of force responses for each state were plotted separately as a function of recipient background forces. Background force values used in the regression plots were the recipient muscle force means for the 15 msec period prior to stretch onset for each stretch repetition. However, if the recipient muscle background force did not change during tibial nerve stimulation but the donor muscle did, the donor background force values were used instead (Bonasera and Nichols 1994). The plotted force responses were fit with least squares quadratic polynomial curves and 95% confidence intervals. An F test using full and reduced polynomial regression models were used to evaluate whether the state 1 and state 2 force responses could be fit with the same line or were statistically different warranting separate polynomial curve fit lines (Kutner et al. 2005; Ross and Nichols 2009; Lyle et al. 2016). The dependent variable was the stretch evoked force responses from the recipient muscle. The full model predictor variables included a grouping variable (state 1 and state 2), background force, background force squared, background force x grouping variable, and background force squared x grouping variable. The reduced model lacked the grouping variable terms. The F test evaluated the null hypothesis that the population of recipient force responses from state 1 and state 2 are the same and should be fit with a single polynomial fit line. In contrast, a *P* value < 0.05 from the F test indicates a significant separation of the force populations suggesting intermuscular spinal pathways from the donor muscle influenced recipient motor output. In addition to testing for significant separation with the F test, the relative magnitude of any difference between the polynomial curve fit vectors from states 1 and 2 were expressed as a percent difference [((state 2 curve fit vector – state 1 curve fit vector)/state 1 curve fit vector) *100].

## Results

The PLANT‐to‐TA tendon transfer remained intact in all four cats that had PLANT transfer with partial TA resection (TT, Table 1). The tendon‐to‐tendon connection was intact but thin in one out of these four cats. Of the four cats with PLANT tendon transfer surgery with deep peroneal nerve transection (TT‐N), only one cat had a thin tendinous connection that remained intact. The other three cats from the TT‐N group exhibited tendon‐to‐tendon failure despite similar restrictions in their activity. These results indicate a limited success of the tendon transfer surgery, particularly in the presence of concurrent transection of the nerve to the pretibial flexors. Nonetheless, the tendon‐to‐tendon ruptures effectively functioned as tenotomies, providing an additional opportunity to evaluate structural adaptations of the musculoskeletal system and spinal circuitry.

### Transferred muscle‐tendons exhibited regrowth often toward original insertion site

**Figure 2:**
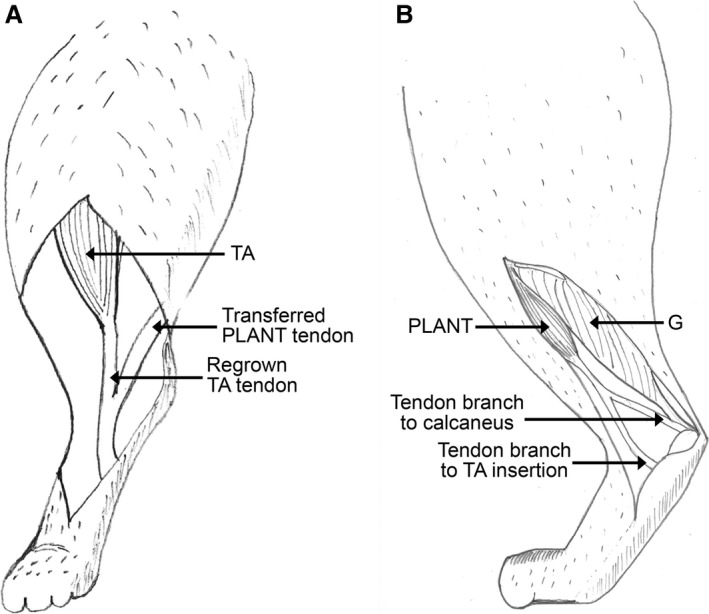
Cartoons illustrating the most common musculotendon adaptations observed during the terminal experiment. (A) TA regrowth to its original insertion joining the transferred PLANT tendon (ventral view) and (B) PLANT regrowth of tendinous connections towards the calcaneus (medial view) TA, tibialis anterior; G, gastrocnemius; PLANT, plantaris.

Whether the tendon‐to‐tendon connection remained intact or tore, the transferred PLANT tendons and resected TA exhibited structural adaptations. The PLANT normally functions primarily as an ankle extensor and plantar flexor of the toes due to its distal insertion to the calcaneus and flexor digitorum brevis (Crouch 1969; Goslow et al. 1972; English and Letbetter 1982). The tendon transfer surgery aimed to change the mechanical action of the PLANT by relocating its distal insertion to the first metatarsal via the TA tendon. Anatomical dissection revealed newly formed connections between the transferred PLANT or resected TA and the skeleton (Fig. 2A and B). Regenerated connections were often toward the original location of its insertion. This was found for six of the PLANT and five of the TA muscles (Table 1). For the transferred PLANT, such tendinous connections were in addition to the intact PLANT‐to‐TA tendon linkage in four cats. These results indicate that the muscle‐tendon tissue response to the agonist‐to‐antagonist tendon transfer was to restore the original connections of the tendons to the skeleton. In the case of the transferred PLANT, this would compromise the aimed new mechanical action of this muscle. In case of the resected and regrown TA tendon, this allowed the TA muscle fibers to transmit forces to the foot (Video S1).

### Muscle masses

Masses of the transferred PLANT muscles were lower than that of the intact left PLANT in all but one cat (TT1) that exhibited equal PLANT masses bilaterally. The range of differences varied between 1.9 and 4.5 g. This was accompanied by compensatory changes in some of the other ankle plantar flexion muscles (Table 2). The TA masses were smaller on the surgical right side in all cats (range 1.3–4.3 g). In the TT group, a compensatory increase in EDL muscle mass was observed, whereas in the TT‐N group the right EDL mass was also decreased as would be expected.

**Table 2:** Mass of several muscles of the cat lower hindlimb following right sided PLANT‐to‐TA tendon transfer

	TT group			TT‐N group		
Mass (g)	Right	Left	T‐test (one‐tail)	Right	Left	T‐test (one‐tail)
PLANT	4.9 ± 2.4	7.2 ± 0.7	*P *=* *0.050	3.6 ± 1.0	7.3 ± 0.4	*P *=* *0.002
G	23.1 ± 2.5	23.1 ± 2.9	*P *=* *0.467	24.6 ± 0.3	22.7 ± 1.1	*P *=* *0.015
SOL	4.0 ± 0.5	3.7 ± 0.3	*P *=* *0.032	4.1 ± 0.9	3.9 ± 0.6	*P *=* *0.157
FHL	5.2 ± 0.6	5.3 ± 1.0	*P *=* *0.234	5.0 ± 0.4	5.0 ± 0.2	*P *=* *0.310
TA	3.2 ± 1.1	5.8 ± 0.7	*P *=* *0.018	2.3 ± 0.6	6.2 ± 0.4	*P *<* *0.001
EDL	3.6 ± 0.6	3.3 ± 0.3	*P *=* *0.033	3.2 ± 0.2	3.7 ± 0.4	*P *=* *0.034

The TA muscle was partly resected in four cats (TT group) and in the other four cats the nerve to the TA and EDL was additionally transected during the transfer surgery (TT‐N group).

PLANT, plantaris; G, gastrocnemius; SOL, soleus; FHL, flexor hallucis longus; TA, tibialis anterior; EDL, extensor digitorum longus.

### Intermuscular interactions from PLANT onto FHL and G were not altered after tendon transfer

For all intermuscular reflex results, cat TT3 was excluded from analyses due to incomplete data secondary to unexplained death during testing. Thus, reflex data will be reported with a sample of three cats from the TT group and four cats from the TT‐N group.

In control cats, the PLANT is known to evoke inhibitory force feedback onto FHL and G (Nichols 1994), whereas TA is known to evoke length‐dependent reciprocal inhibition onto FHL and G (Nichols 1994, 1999). If the intermuscular circuitry from PLANT reorganized to be like that of the TA, it would be expected that inhibition evoked from PLANT, if present, would be characteristic of length‐dependent reciprocal inhibition and thus lack force‐dependent effects. This possibility was evaluated by comparing the left control and right transferred muscle pairs within cats. It should be noted that the relative magnitude of intermuscular inhibition can be influenced by the background forces achieved during the crossed extension reflex in recipient muscles (i.e., motor units available to derecruit), and in the case of force feedback also the donor muscles (Bonasera and Nichols 1994).

In the group of seven cats with reflex data, the crossed extension reflex elicited a background force change in only two out of seven of the right transferred PLANT muscles as donor (i.e., TT1, TT4). This observation, if coupled with PLANT responding to ipsilateral tibial nerve stimulation (i.e., flexion withdrawal reflex characteristic of TA), would support spinal circuit reorganization. However, there was never a PLANT force increase to ipsilateral stimulation (i.e., flexion withdrawal reflex), and the left control PLANT muscles as donor also did not respond in four out of seven cats. While it is unknown why the PLANT muscles did not consistently respond to the crossed extension reflex, the available data suggest the lack of response is not due to spinal circuit reorganization. Nonetheless, it should be noted that the lack of PLANT background force modulation is likely to have resulted, if anything, in an underestimate of the relative magnitudes of inhibition observed.

**Figure 3:**
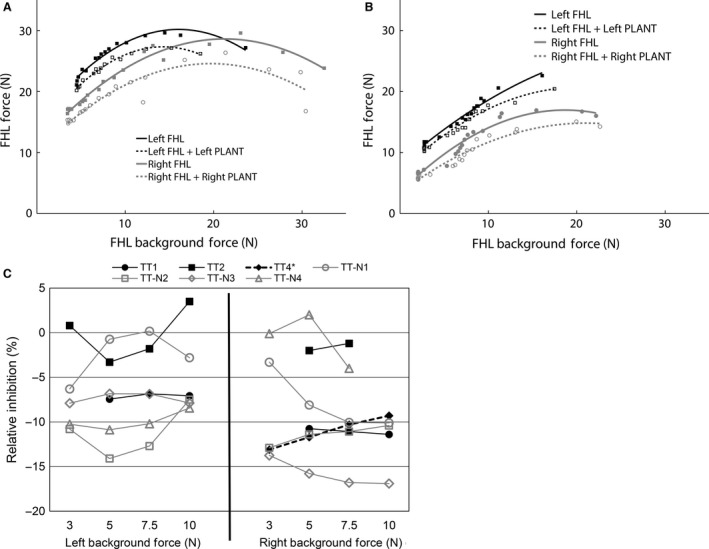
Examples of significant inhibition from PLANT onto FHL from cats in which the tendon transfer tendon‐to‐tendon connection (A) remained intact (TT1), or (B) tore (TT‐N3, tendon transfer tore with thin tissue band in continuity and regrowth of tendinous band from PLANT to calcaneus). (C) Relative inhibition from PLANT onto FHL for all cats at 3, 5, 7.5, and 10 N of background force. Significant inhibitory feedback was observed for all cats (*P *<* *0.001) except for TT2 and TT‐N4 right. *cat TT4 had data for the right limb only. FHL, flexor hallucis longus; PLANT, plantaris

The interaction from PLANT onto FHL was evaluated during the crossed extension reflex in three cats from the TT group (TT1, TT2, TT4 with data available for right side only) and all four cats from the TT‐N group. There was significant inhibitory feedback observed from PLANT onto FHL in two out of three cats from the TT group (bilateral TT1 and right side TT4, all *P *<* *0.001). Significant inhibition from PLANT onto FHL was observed in all four cats that underwent tendon transfer with deep peroneal nerve transection (bilateral in three cats *P *<* *0.001; cat TT‐N4 *P *<* *0.001 on left side only). Examples of inhibitory feedback from PLANT onto FHL on the right and left limbs from each group are shown in Figure 3. It can be seen that the relative inhibition from PLANT onto FHL on the right transferred side and left control side was similar whether the tendon repair was successful (Fig. 3A, TT1) or unsuccessful (Fig. 3B, TT‐N3). A summary of the relative inhibitory magnitudes observed from PLANT onto FHL in all subjects at 3, 5, 7.5, and 10 N of background force is shown in Figure 3C. The background force values were chosen to account for the range common to most cats. The inhibitory feedback observed from the left control and right transferred PLANT onto FHL ranged from 5% to 17%. As noted above, inhibitory feedback from the right transferred PLANT onto FHL was observed whether the tendon repair remained intact (e.g., TT1), partially intact (e.g., TT4, TT‐N3) or was completely torn (e.g., TT‐N1, TT‐N2). While not a universal finding, a force‐dependent inhibitory interaction can be seen on the right transferred side in some cats (e.g., TT‐N1 and TT‐N3), which is a feature characteristic of Golgi tendon organ feedback (Nichols 1999).

**Figure 4:**
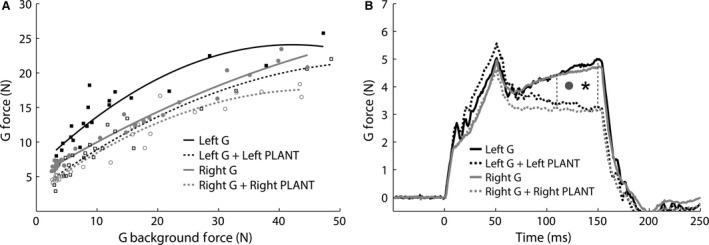
Significant inhibitory feedback from PLANT onto G in cat TT‐N3 during the (A) crossed extension reflex (*P *<* *0.001 for left and right), and (B) the resting state with constant background force, that is, no crossed extension reflex (*P *<* *0.001 for left (asterisk) and right (grey circle)). G, gastrocnemius; PLANT, plantaris

The interaction from PLANT onto G was evaluated in four out of seven cats (TT1, TT‐N2, TT‐N3, TT‐N4) during the crossed extension reflex. Significant inhibition was found bilaterally from PLANT onto G in two cats (TT‐N2 and TT‐N3, both *P *<* *0.001). The intermuscular inhibition was larger at higher background forces bilaterally in both cats which is characteristic of Golgi tendon organ feedback (Nichols 1999), and illustrated in Figure 4A for cat TT‐N3. In the other two cats (TT1, TT‐N4), no PLANT onto G interaction was observed on the right or left limbs. We interpret the lack of PLANT onto G effect bilaterally to indicate that this intermuscular interaction was not conducive to evaluate in these two cats (e.g., central suppression) and not evidence for change in circuitry. For example, strong inhibitory feedback from PLANT onto FHL was observed in cat TT1 (see Fig. 3A).

The PLANT onto G interaction was also evaluated during constant background forces (i.e., not elicited by the crossed extension reflex) in four cats (TT‐N1, TT‐N2, TT‐N3, TT‐N4). Significant inhibitory feedback from PLANT onto G of similar magnitude for the right and left limbs was observed in three cats (TT‐N2 right 36% vs. left 32% inhibition, both sides *P *<* *0.001; TT‐N3 right 31% vs. left 30% inhibition, both sides *P *<* *0.001; TT‐N4 right 10% vs. left 18% inhibition, both sides *P *<* *0.001). Mean force response profiles illustrating the inhibitory interaction for cat TT‐N3 are shown in Figure 4B. Cat TT‐N1 exhibited no inhibition from PLANT onto G bilaterally, and we interpret this finding the same as that described above. Taken together, these results suggest tendon transfer surgery, which remained intact, partially torn or completely torn and resulted in significant atrophy of PLANT (Table 2), did not change the organization or the relative inhibitory magnitude of proprioceptive feedback from PLANT onto FHL and G.

### Transferred PLANT received inhibitory force feedback typical of control animals

The FHL typically evokes strong inhibitory force feedback onto PLANT (Ross and Nichols 2009), and weak length‐dependent reciprocal inhibition onto TA at best (Nichols 1989; Nichols et al. 1999). Thus, if PLANT spinal reflex circuitry reorganized to be like that of TA, then intermuscular actions from the FHL onto PLANT would be expected to exhibit weak inhibitory effects.

**Figure 5:**
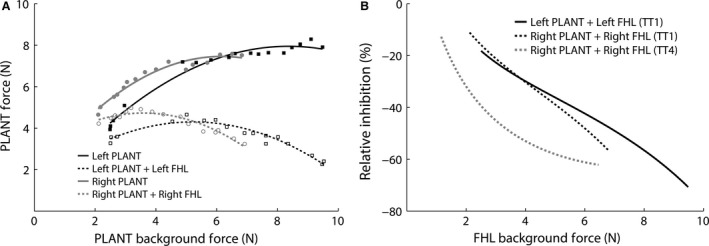
(A) Significant force‐dependent inhibition from left and right FHL onto PLANT (TT1). (B) Relative inhibition from FHL onto PLANT in cats TT1 and TT4. It can be seen that the relative inhibition increased at higher background force. *Cat TT4 had data available for the right transferred side only. PLANT, plantaris; FHL, flexor hallucis longus.

Evidence in favor of preserved spinal circuitry after tendon transfer surgery was found in two cats from the tendon transfer with TA resection group. In both cats (TT1, TT4 with data available for right side only), FHL evoked strong force‐dependent inhibition onto PLANT on the right transferred side (*P *<* *0.001, Fig. 5A,B) characteristic of Golgi tendon organ feedback. The remaining cat from the TT group (TT2) and all four cats from the transfer with concurrent pretibial flexor denervation group exhibited smaller (TT‐N2: 10–20% inhibition, *P *<* *0.01) or negligible (TT2, TT‐N1, TT‐N3, TT‐N4: 0–10% inhibition at best) inhibition compared to cats TT1 and TT4. The most parsimonious explanation for the smaller or negligible inhibition in these cases was lack of the PLANT responding to the crossed extension reflex (i.e., no background force change) in all five cats (TT2, TT‐N1, TT‐N2, TT‐N3, TT‐N4). In these cats, the steady state PLANT background force and their associated stretch induced force responses provided a more limited and constant motor unit pool available for FHL afferent feedback to derecruit compared to cats TT1 and TT4. Taken together, these findings indicate the surgically transferred PLANT muscles, whether the repair remained intact (e.g., TT1) or was unsuccessful (e.g., TT‐N2), received inhibitory feedback most consistent with that expected under control conditions.

### Evidence for preservation of reciprocal inhibition from EDL onto PLANT and G

Typically, the pretibial flexors (i.e., EDL and TA) exchange length‐dependent excitation and evoke length‐dependent reciprocal inhibition onto the ankle extensor muscles (e.g., G and PLANT) (Nichols 1989). If intermuscular spinal reflex circuitry from PLANT reconfigured to be typical of TA, then it would be expected that the usual reciprocal inhibition evoked from EDL onto PLANT would be replaced with excitatory feedback.

**Figure 6:**
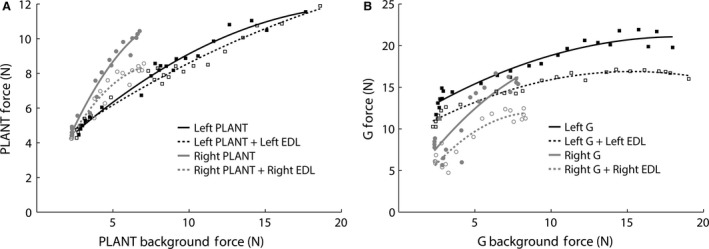
(A) Significant reciprocal inhibition was found from extensor digitorum longus (EDL) onto the transferred PLANT in cat TT1 (right *P *<* *0.001, left *P *=* *0.02) suggesting the transferred PLANT retained its usual intermuscular reflex interaction. If PLANT circuitry reorganized to assume the transferred actions of TA, then EDL would have evoked excitatory feedback onto PLANT. (B) Significant reciprocal inhibition was also observed from EDL onto G in cat TT1. PLANT, plantaris; EDL, extensor digitorum longus; G, gastrocnemius

The interaction between EDL and PLANT was evaluated in only one cat (TT1) from the TT group. It was found that EDL significantly inhibited PLANT bilaterally but the effect was small on the intact left side (Fig. 6A, right *P *<* *0.001, left *P *=* *0.02). Weak inhibitory feedback from PLANT onto EDL was also observed bilaterally (*P *<* *0.001, data not shown). These results are consistent with prior reports finding larger reciprocal inhibition from pretibial flexors to the extensors than vice versa (Nichols 1989; Nichols and Koffler‐Smulevitz 1991). In addition, significant reciprocal inhibition from EDL onto G was observed (Fig. 6B, right and left *P *<* *0.001). While the interaction between EDL and G was not expected to change due to the surgical intervention, we include this data as further support and context for preservation of spinal reflex circuitry after tendon transfer surgery.

### Evidence for preservation of reciprocal inhibition evoked by the tenotomized and regenerated TA muscle tendon

**Figure 7:**
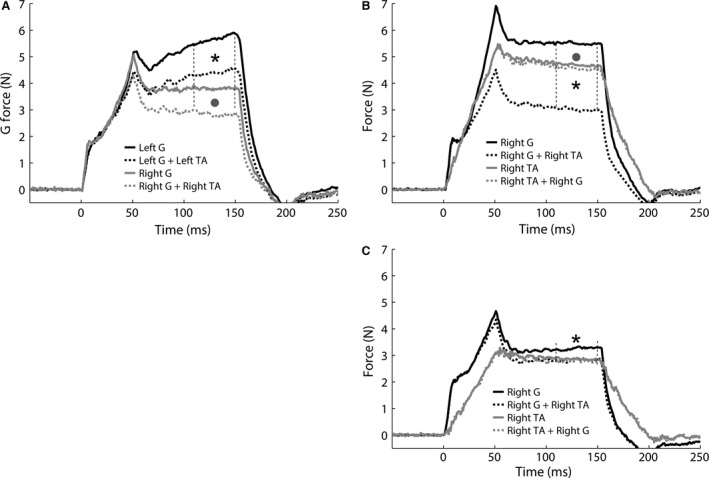
(A) Example of similar reciprocal inhibition from the intact left and transected and regenerated right TA tendon onto G in cat TT2 (*P *<* *0.001 for left (22% asterisk) and right (25% grey circle)). (B, TT4) and (C, TT‐N4): Examples of preserved reciprocal inhibition from transected and regenerated TA onto G with stronger inhibition from TA onto G (TT4: TA onto G 45%, asterisk *P *<* *0.001; G onto TA 3%, grey circle *P *<* *0.001; TT‐N4: TA onto G 13%, asterisk *P *=* *0.002; G onto TA 0%, *P *=* *0.12). It should be noted that the deep peroneal nerve was cut in (C) and thus the inhibitory feedback from TA onto G indicates self‐reinnervation occurred. The stretch reflex is permanently lost in muscles after self‐reinnervation. The reduced slope in the TA stretch evoked force response in (C) (grey lines) is a characteristic feature of the lost stretch reflex (Cope et al. 1994; Huyghues‐Despointes et al. 2003; Lyle et al. 2016). G, gastrocnemius; TA, tibialis anterior.

It has been previously shown that TA and G exchange reciprocal inhibition with stronger inhibitory feedback from TA onto G than vice versa (Nichols 1989). The fate of the reciprocal inhibitory circuitry was evaluated in two cats in which the TA regrew to its original insertion after transection and partial resection (TT2, TT4) and in one cat with additional deep peroneal nerve transection (TT‐N4). The interaction from TA onto G was evaluated bilaterally in cat TT2 and on the right side only in cats TT4 and TT‐N4. Significant inhibition was found bilaterally in cat TT2 (Fig. 7A, left 22% vs. right 25%, *P *<* *0.001 for both). Shown in Figures 7B&C are additional findings consistent with that observed in control animals illustrating stronger inhibitory feedback from right TA onto G compared to that evoked from G onto TA (TT4: TA onto G 45%, *P *<* *0.001; G onto TA 3%, *P *<* *0.001; TT‐N4: TA onto G 13%, *P *=* *0.002; G onto TA 0%, *P *=* *0.12). The finding of reciprocal inhibition from TA onto G in cat TT‐N4 was initially surprising given the nerve to TA was transected without surgical repair (i.e., the intent was to permanently denervate the pretibial flexors). This observation highlights the capacity for nerve regrowth and the need to place a physical barrier on the nerve stump to prevent reinnervation. The preservation of reciprocal inhibition in the self‐reinnervated TA is compatible with our recent findings that intermuscular sensory feedback can regain functional connectivity after self‐reinnervation (Lyle et al. 2016). Moreover, the reduced slope in the TA force response during the 50 msec ramp stretch in Figure 7C suggests loss of the stretch reflex, a characteristic finding of self‐reinnervated muscle (Cope et al. 1994; Huyghues‐Despointes et al. 2003; Lyle et al. 2016). Collectively, these data provide evidence for preservation of length‐dependent sensory feedback after tenotomy and subsequent regrowth of tendons, as well as after self‐reinnervation.

## Discussion

The purpose of this study was to examine the possibility that the unsatisfactory functional outcomes after agonist‐to‐antagonist tendon transfer are due to failure of the muscle to effectively function in its new role either mechanically due to myofascial connections and/or neurally because intermuscular spinal reflex circuitry fails to reorganize. This is the first study investigating intermuscular reflex pathways following tendon transfer and associated musculotendon adaptations. While several experimental challenges were encountered, the major outcomes of this work were: (1) new tendinous connections were formed following PLANT‐to‐TA tendon transfer, which often inserted near their original location of insertion on the skeleton; (2) irrespective of the outcome of the PLANT‐to‐TA tendon transfer (i.e., intact or torn), the intermuscular spinal reflex interactions between PLANT and synergistic ankle plantar flexors and between PLANT and antagonistic ankle dorsal flexors were unaltered, which was also the case for the transected and regenerated TA. These primary findings suggest that patient outcomes after tendon transfer surgery could be compromised by both musculotendon plasticity and lack of intermuscular spinal reflex circuitry adaptation.

### Robustness of spinal circuitry to changes of musculoskeletal organization

We initially hypothesized that, after tendon transfer, a potential mismatch between the pattern of motor output and the new pattern of sensory feedback could induce reorganization of local sensory circuitry. However, we did not find any evidence of changes in intermuscular spinal reflex pathways in response to the changes in the organization of the musculoskeletal system. These findings are in general agreement with previous literature on tendon transfers in which no or limited changes in muscle activation patterns have been reported during movement (locomotion, grasping) in human patients as well as different animal models (e.g., rat, cat), including neonatal animals (Sperry 1945; Leffert and Meister 1976; Forssberg and Svartengren 1983; Illert et al. 1986; Loeb 1999; Slawinska and Kasicki 2002; Maas et al. 2010b; Van Heest et al. 2010). These general findings also appear to parallel a general lack of central reorganization reported after cross‐reinnervation of various hindlimb muscles in the cat (Eccles et al. 1962; Luff and Webb 1985; O'Donovan et al. 1985; Gordon et al. 1986). Taken together, a mismatch between mechanical action and local sensory feedback does not appear to be a robust stimulus to induce central reorganization of pattern generator or intermuscular sensory feedback circuitry.

The lack of spinal reflex reorganization observed in this study could be attributed to several alternative explanations. We did not assess muscle fascicle length changes or EMG activity during locomotion as in some of our previous studies (Maas et al. 2009, 2010a, 2010b). Therefore, the presence of a mismatch between muscle activation and sensory feedback due to the new mechanical action of the muscle cannot be confirmed. However, it should be noted that we did confirm that dorsiflexion resulted in PLANT muscle shortening at the time of surgery, and we confirmed in several cases (*n *=* *4, TT2, TT3, TT4, TT‐N3) at the terminal experiments that the PLANT was shortened upon ankle dorsiflexion (Video S1). We propose it is very likely that the usual temporal feedback from muscle spindles and tendon organs were affected by the surgical intervention. Nonetheless, a lack of stimulus for reorganization could have resulted from the musculotendon plasticity observed (see below). In addition, the PLANT tendon transfer was successful in only five out of eight cats and in some cases the muscle belly was substantially atrophied and the tendon thinner than normal to cover the distance between origin and insertion. We conclude that despite the significant and varied changes to the musculoskeletal system observed, the lack of changes in reflex pathways indicates that changing the anatomy of the musculoskeletal system and, hence, sensory feedback, did not provide a robust signal for changes in the organization of sensory pathways in the spinal cord in this sample, as concluded previously (Forssberg and Svartengren 1983). In support of our findings, others have found that tenotomy and immobilization at a shortened length, both associated with muscle atrophy, does not affect the number of muscle receptors and causes limited changes in their morphology and stretch responses (Jozsa et al. 1988; Nordstrom et al. 1995). However, it also remains possible that extensive rehabilitation and precluding additional tendinous connections to adjacent tissues after tendon transfer could result in spinal reflex reorganization.

### Tissue adaptation and muscle mechanics following tendon transfer

In most patients, orthopedic surgical interventions are performed only once. The surgeon can thus not directly evaluate the anatomy following a tendon transfer, and must use indirect outcome measures. Commonly, the changes in joint range of movement or limb function are assessed (e.g., Kreulen et al. 2006; van Alphen et al. 2013; Van Heest et al. 2015). Occasionally, the changes in muscle activation patterns (Leffert and Meister 1976; Illert et al. 1986; Van Heest et al. 2010) and, sporadically, the actual mechanical effect of the transferred muscle is assessed (Riewald and Delp 1997).

When we exposed the lower leg and examined the transferred PLANT and transected TA, we found a variety of adaptations, but two outcomes were found in the majority of cats: (1) additional tendinous connections from PLANT towards the calcaneus, (2) regeneration of the transected TA tendon and torn PLANT tendon towards the original location of the insertion on the foot. Regarding the second observation, tendon regeneration (“reunion”) has been found to generally occur after harvesting the tendon of either semitendinosus or gracilis for anterior cruciate ligament reconstruction (e.g., Ahlen et al. 2012; Nakamae et al. 2012), as observed using magnetic resonance imaging (MRI) and three‐dimensional computed tomography (3DCT). Also following tenotomy of hamstrings for improvement of knee extension in children with spastic cerebral palsy, tendon regeneration has been observed during repeat operation (Dhawlikar et al. 1992). Although the phenomenon of tendon regeneration has been described frequently, the exact mechanisms are not yet fully understood (Otoshi et al. 2011; Janssen et al. 2013). In any case, similar to the studies described above, the transected TA tendon was found to regenerate in five of eight cats in this study. The fact that the anterior crural compartment is rather tight and there are no other options for growth may explain why the regenerated TA tendon was located very near its original position. In agreement with this, the path of the regenerated torn PLANT tendon was much more variable. Importantly, the regenerated TA muscle‐tendon complex still evoked reciprocal inhibition as generally observed in control cats. We speculate that a similar finding would be observed in regenerated hamstring tendons after anterior cruciate ligament reconstruction.

Even when the PLANT‐to‐TA tendon transfer was successful (five out of eight cats), additional tendinous connections in the direction of the calcaneus were observed. This suggests that the mechanical effect of PLANT was not fully changed from ankle plantar flexion to dorsal flexion. Following a tendon transfer to the flexor site of the knee in subjects with spastic cerebral palsy, the rectus femoris (a knee extensor) has been reported to still exert an extension moment at the knee held at 90° flexion (Riewald and Delp 1997). A similar result was recently obtained after transferring the flexor carpi ulnaris (FCU) to the extensor carpi radialis insertion in rats if tested at extended wrist positions (Maas and Huijing 2012b). If tested at flexed wrist positions, however, FCU did exert a wrist extension moment according to its new insertion. Additional experiments indicated that the transferred FCU was tightly linked to surrounding structures via newly formed connective tissues (Maas and Huijing 2012a) that were capable of transmitting force to neighboring structures, such as muscles acting as agonists prior to the tendon transfer (Maas and Huijing 2011; Maas et al. 2013). Based on our anatomical observations in this study, we deem it likely that the transferred PLANT also had a bidirectional mechanical effect at the ankle joint (i.e., exerting a plantar flexion moment at dorsi‐flexed ankle positions, but dorsal flexion moments at plantar‐flexed ankle positions).

### Limitations

It appeared not to be possible to change the mechanical effect of PLANT to a clean dorsal flexion moment at the ankle joint, compromising the original aim of the study. The PLANT‐to‐TA tendon tore in three cats and the intact tendon‐to‐tendon connections were thin in three of five successful cases in this study. Aside from the surgical transfer, we observed musculotendon growth from the transferred PLANT and the transected TA. The attempt to prevent recurrence of TA function by denervation, which was intended to force the animal to use the transferred PLANT for ankle dorsal flexion, resulted in even worse results. This can probably be explained by a more extended ankle position, as found previously (Carrier et al. 1997), resulting in more muscle‐tendon unit strain of transferred PLANT. A comprehensive mechanical assessment of musculotendon adaptations would have been ideal, but it was simply not feasible due to the time needed for the spinal reflex testing at the terminal surgery.

The tearing and stretching of the PLANT‐to‐TA tendon‐tendon connections may have been the result of our choice to use their housing conditions as a way to “restrict” activity level, as opposed to brace immobilization. The goal was to limit jumping and running for 8 weeks by keeping them in pairs separate from the colony, but the cats still had the ability to do as they chose in the cage or room. As such, we favor movement related tendon stress as the reason for tendon‐to‐tendon failures over surgical technique given the tendon repair method used in this study (i.e., Pulvertaft) is widely used in human tendon transfer procedures (Kulikov et al. 2007; De Smet et al. 2008; Fuchs et al. 2011). Our findings strongly suggest brace immobilization would be warranted in future studies of cat hindlimb tendon transfer.

## Conclusion

Although we encountered several challenges regarding the tendon transfer surgery, the preponderance of evidence from this study strongly suggest connective tissue adaptations and lack of neural circuitry plasticity could both present challenges for patients recovering from tendon transfer surgery or other orthopedic interventions affecting skeletal muscles. Specifically, surgical planning and outcomes assessments after tendon transfer surgery should consider potential consequences of the transferred muscle's intermuscular spinal circuit actions.

## Conflict of Interest

No conflict of interest, financial or otherwise, are declared by the authors.

## Data Accessibility

## Supporting information




**Video S1:** Shown is the influence of ankle movement on the transferred plantaris at the time of the terminal experiment. It can be seen that ankle dorsiflexion results in shortening of the transferred plantaris muscle and plantar flexion results in lengthening. These observations are consistent with the intended new mechanical action due to the tendon transfer. Although difficult to see, the tibialis anterior regenerated back to its original insertion despite partial muscle resection in this cat.
