# Supplementary material for: Musculotendon adaptations and preservation of spinal reflex pathways following agonist‐to‐antagonist tendon transfer
Source: Physiol Rep. 2017 May 3;5(9):e13201. doi: 10.14814/phy2.13201 (PMC5430118; doi:10.14814/phy2.13201)
Supplement: Supplementary file 2 [file PHY2-5-e13201-s002.docx]

Shown is the influence of ankle movement on the transferred plantaris at the time of the terminal experiment. It can be seen that ankle dorsiflexion results in shortening of the transferred plantaris muscle and plantar flexion results in lengthening. These observations are consistent with the intended new mechanical action due to the tendon transfer. Although difficult to see, the tibialis anterior regenerated back to its original insertion despite partial muscle resection in this cat.
